# Preparation Process and Properties of Pellets from PFC1 Hematite Concentrate

**DOI:** 10.3390/ma19194138

**Published:** 2026-09-28

**Authors:** Yong-Hong Wang, Ping Du, Xu-Xu Ma, Wei Zhang

**Affiliations:** 1 Institute Research of Iron & Steel of Sha-Steel, Zhangjiagang 215625, China; 2 Jiangsu Key Laboratory of New Energy Steel and Green Manufacturing, Zhangjiagang 215625, China

**Keywords:** PFC1 hematite concentrate, ore blending, firing characteristics, metallurgical properties

## Abstract

The shortage of high-quality pellet feed has constrained the green transformation of blast furnaces, making it urgent to find economical alternative materials. This study investigated the effects of different blending ratios (0–30%) of Brazilian hematite concentrate PFC1 on the ballability, firing characteristics, and metallurgical properties of the mixed feed. The results showed that PFC1 had a relatively coarse particle size (59.1% passing −0.074 mm) and a low specific surface area (847 cm^2^/g), but exhibited a rapid capillary water migration rate (13.33 mm/min) and an optimal static ballability index (K_p_ = 0.26). At PFC1 blending ratios of 10–20%, the green balls achieved a drop strength >4 times/0.5 m, a compressive strength of approximately 18 N·P^−1^, and a decrepitation temperature ≥ 480 °C, demonstrating the best balling performance. In firing tests, the pellet strength showed a decreasing trend with increasing PFC1 proportion; however, under the firing regime of 1220 °C for 9–15 min, the compressive strengths of finished pellets from all schemes exceeded 2700 N, meeting the requirements for blast furnace use. The addition of PFC1 significantly improved the reducibility (RI increasing from 52% to 66.4%), while the reduction swelling index remained controllable (RSI ≤ 12.44%). Pot-grate tests validated the laboratory results, and the recommended industrial blending ratio of PFC1 is 10–20%. These findings provide a theoretical basis and process reference for the economical utilization of hematite-type concentrates.

## 1. Introduction

The shortage of high-quality pellet feed has become a critical bottleneck restricting the increase in pellet proportion in blast furnace burdens and the transition from sintering to pelletizing. Pellets, characterized by uniform particle size, high strength, excellent metallurgical properties, as well as significantly lower process energy consumption and carbon emissions compared with sinter [1,2,3], have made their increased proportion in blast furnace burdens an important direction for green and low-carbon development in the ironmaking industry. However, domestic magnetite resources are characterized by low grade and high processing costs, while the prices of imported high-quality magnetite concentrates continue to rise, necessitating the urgent search for economically feasible alternative raw materials [4]. PFC1 concentrate, a Brazilian hematite product featuring high iron grade, low gangue content, and notable cost advantages, represents a potential pathway for replacing a portion of traditional magnetite concentrates.

The consolidation mechanisms of hematite and magnetite are fundamentally different [5,6,7]: magnetite oxidizes during firing to generate highly reactive secondary Fe_2_O_3_, which promotes recrystallization bonding; whereas primary hematite particles are difficult to recrystallize, requiring higher temperatures and longer holding times, and single hematite concentrate exhibits poor ballability. During oxidation, magnetite predominantly transforms into maghemite (γ-Fe_2_O_3_), whose freshly formed Fe_2_O_3_ exhibits high reactivity; thus, appropriate blending of magnetite concentrate can effectively improve pellet quality by utilizing this oxidation product. To this end, this study systematically investigates the effects of PFC1 blending ratio on the ballability, firing characteristics, and metallurgical properties of the blended concentrates, aiming to provide technical basis and theoretical support for its industrial application.

## 2. Raw Material Properties and Experimental Methods

### 2.1. Chemical Composition

The experimental raw materials included three iron ore concentrates and one bentonite, and their main chemical compositions are presented in Table 1. The chemical compositions of all raw materials were quantitatively analyzed using a wavelength-dispersive X-ray fluorescence spectrometer (ZSX Primus III NEXT or ZSX Primus II, Rigaku, Akishima, Japan) equipped with a 3 kW or 4 kW Rh target X-ray tube. Prior to analysis, the samples were dried in an oven at 105 °C to constant weight, and then glass fusion beads were prepared by the high-temperature fusion method to eliminate the influence of mineralogical effects and particle size effects on the analytical results. The elemental contents are reported in the form of oxides, and the loss on ignition (LOI) was determined by the high-temperature gravimetric method.

The physicochemical properties of bentonite have a significant influence on the quality of green pellets. To comprehensively evaluate the quality of the bentonite used in this study, its key performance indicators were systematically determined in accordance with GB/T 20973-2020 [8] bentonite. The montmorillonite content of the bentonite was indirectly determined by the methylene blue index method, expressed as the grams of anhydrous methylene blue adsorbed per 100 g of bentonite, the cation exchange capacity (CEC) was measured using the ammonium chloride–ethanol method, with the results expressed as the total exchangeable cation content (mmol/100 g). The swelling capacity was determined by the volumetric method, i.e., the volume expansion of 1 g of bentonite after standing in hydrochloric acid solution for 24 h (mL/g). The colloid index was measured by the volumetric method, in which 15 g of bentonite was dispersed in water and allowed to stand for 24 h, and the gel volume (mL/15 g) was recorded. The water absorption rate of bentonite was determined by the glass capillary method, recording the natural water absorption capacity of the sample at 20 °C and 0.1 MPa for 2 h. All measurements for each indicator were performed in parallel, and the average values were reported as the final results.

As shown in Table 1, BL concentrate is a typical magnetite concentrate, with a gangue (SiO_2_ + Al_2_O_3_) content of only 2.48%, but with a relatively high sulfur content. AZ concentrate is also a magnetite concentrate, with an Al_2_O_3_ content of only 0.25%, yet its SiO_2_ content is as high as 8.17%, and the alkali metal (Na_2_O + K_2_O) content is relatively high (0.32%). In contrast, PFC1 is a typical hematite, with an iron grade of 62.45%, SiO_2_ content of 5.52%, and low levels of Al_2_O_3_ and harmful elements. However, its loss on ignition reaches 2.92%, which may lead to aggravated decrepitation of green balls after blending, and the strength of finished pellets after firing may also decrease to varying degrees. The bentonite contains a relatively high SiO_2_ content, which will introduce more impurities upon usage and reduce the pellet grade.

To further determine the incorporation states of chemical constituents within the mineral lattice, the three iron ore concentrates were characterized by X-ray diffraction (XRD) using a Malvern Panalytical Aeris benchtop diffractometer (dedicated configuration for metallic and mining materials). Diffraction patterns were acquired with Cu Kα radiation (λ = 1.5406 Å) at 40 kV and 40 mA, over a 2θ range of 10–90° with a step size of 0.02°. The results are shown in Figure 1.

The XRD phase results are in good agreement with the chemical compositions. Both BL and AZ concentrates feature magnetite (Fe_3_O_4_) as the dominant phase, with distinct characteristic peaks. The AZ sample exhibits considerably stronger quartz diffraction than the BL sample, corroborating its higher silica content. In contrast, the PFC1 sample shows a distinctly different phase assemblage, predominantly hematite (Fe_2_O_3_) and goethite (FeO(OH)), with minor quartz, consistent with a limonitic–hematite type concentrate.

### 2.2. Particle Size Distribution

The particle size distributions of the three iron ore concentrates were determined using a Winner 2308A laser particle size analyzer (Jinan Winner Particle Instrument Co., Ltd., Jinan, China), and the results are presented in Table 2. The measurements were performed by the dry dispersion method, using compressed air as the dispersion medium at a dispersion pressure of 0.3 MPa. The feed rate was controlled within the range of 30–50% via a vibratory feeder, covering a measurement range of 0.05–2000 μm. The obscuration was maintained between 5% and 15% during each measurement to ensure an optimal signal-to-noise ratio. All samples were tested in triplicate, and the average values were reported. The instrument was calibrated using standard reference materials before each test session, and the measurements conformed to ISO 13320:2020 [9] (Particle size analysis—Laser diffraction methods).

The particle size distributions of the three iron ore concentrates are presented in Table 2. Significant differences in particle size distribution were observed among the three concentrates. BL concentrate was predominantly fine, with 93.3% passing 0.074 mm, of which 60.52% was ultrafine particles (<0.038 mm). The intermediate size fractions of 0.045–0.074 mm and 0.038–0.045 mm accounted for 19.58% and 13.20%, respectively, indicating a “fine-dominant, broadly graded” characteristic with a well-balanced arrangement of coarse and fine particles. AZ concentrate exhibited the finest particle size, with 98.9% passing 0.074 mm and 88.9% ultrafine particles (<0.038 mm), while each intermediate size fraction accounted for less than 6%, reflecting an “extremely fine-dominated and narrowly graded” feature lacking coarse particle skeletons. In contrast, PFC1 concentrate was notably coarser, with particles >0.15 mm and 0.074–0.15 mm accounting for 18.66% and 22.24%, respectively, together exceeding 40%, whereas only 59.1% passed 0.074 mm and only 34.06% were ultrafine (<0.038 mm), indicating a severe deficiency in fine particles and an imbalanced particle size distribution.

Based on the particle size characteristics, BL concentrate, with 93.3% passing 0.074 mm and a wide distribution across coarse, intermediate, and fine fractions, was expected to exhibit the best balling performance. AZ concentrate, with 98.9% passing 0.074 mm and 88.9% ultrafine particles, was excessively fine and narrowly graded, thus requiring blending with coarser materials. PFC1 concentrate, with only 59.1% passing 0.074 mm and over 40% coarse particles, suffered from a serious shortage of fines, making it difficult to pelletize alone; therefore, it must be blended with fine concentrates to establish a rational particle size distribution.

### 2.3. Microstructure

To reveal the differences in particle morphology among the three iron ore concentrates and their influence on balling performance, the micro-morphology of the samples was characterized by scanning electron microscopy (SEM) using a Hitachi SU8000 field-emission scanning electron microscope (Hitachi, Tokyo, Japan). For each sample, five fields of view were randomly selected for image acquisition to ensure the representativeness of the observations. The results are shown in Figure 2.

The AZ and BL concentrates are significantly finer than PFC1. They are magnetite ores with dense, smooth, and irregular particles, some exhibiting polyhedral shape; PFC1 particles are also irregular but more varied—dense smooth flakes are specularite, smooth granules are likely martite, and rough granules are goethite. Fine magnetite particles offer large specific surface area and strong capillary forces, favoring dense green balls [10], and oxidize to active Fe_2_O_3_ during firing to promote consolidation. In contrast, the flaky specularite and smooth granular particles of PFC1 show low surface activity and weak interparticle friction, impairing mechanical interlocking and capillary force retention; rough goethite granules increase friction but their porous structure tends to introduce defects during firing, hindering densification and strength development.

### 2.4. Physical Properties

To systematically analyze and compare the balling characteristics of the three concentrates, their key physical properties were determined, and the results are presented in Table 3.

The specific surface area (SSA) of pellet feeds is an important indicator that is believed to have a great impact on their ballability. Here, the specific surface area was measured using the Blaine air permeability method according to the Chinese national standard GB/T 8074-2008 [11], using a Blaine apparatus (DBT-127 model, Wuxi Jianyi Instrument Machinery Co., Ltd., Wuxi, China).

The bulk density of the raw materials was determined using a container of known volume, and was calculated by Equation (1). Each sample was measured in triplicate, and the average value was reported as the bulk density. The true density was measured using a pycnometer method, and was calculated by Equation (2). The void fraction (porosity) of the material bed was then calculated using Equation (3):

(1) $$\rho_{b}=\frac{m}{V}$$

(2) $$\rho_{t}=\frac{m}{V_{s}}$$

(3) $$\varepsilon =\;1-\frac{\rho_{b}}{\rho_{t}}$$

where *ρ*_b_ is the bulk density (g/cm^3^), m is the mass of the sample (g), V is the total volume of the container (cm^3^), *ρ*_t_ is the true density (g/cm^3^), *V_s_* is the true volume of the sample (cm^3^), and *ε* is the bed porosity (%).

The capillary water migration rate was calculated based on the maximum molecular water (W_m_, %) and maximum capillary water (W_c_, %) contents. These two parameters were determined using the filtration method and the volumetric method, respectively, following the procedures outlined in Theories and Technologies of Iron Ore Agglomeration [12]. Prior to the tests, all samples were dried in an oven at 105 °C for more than 4 h. The static ballability index (K_p_) was calculated according to Equation (4):

(4) $$K_{p}=\frac{W_{m}}{W_{c}-W_{m}}$$

A K_p_ value of less than 0.3 indicates poor ballability of the iron ore concentrate, while a value greater than 0.6 indicates good ballability.

To systematically analyze and compare the balling characteristics of the three concentrates, their key physical properties were determined, and the results are presented in Table 3. Among the three concentrates, PFC1 exhibited the lowest specific surface area (847 cm^2^/g) and the smallest bed porosity (54.43%), which are favorable for improving the packing density of green balls. It also showed the highest capillary water migration rate (13.33 mm/min), which can accelerate moisture removal during the drying stage of green balls, thereby enhancing the balling rate and thermal stability of the green pellets. Furthermore, PFC1 achieved the highest static ballability index (K_p_ = 0.26) among the three concentrates, indicating its superior static balling ability. By comparison, the K_p_ values of the BL and AZ concentrates were only 0.11 and 0.16, respectively, both lower than that of the PFC1 concentrate. Overall, the advantages of PFC1 in hydrophilicity and capillary water migration can effectively compensate for its relatively coarse particle size, suggesting that it possesses good balling potential when blended with appropriate fine fractions.

### 2.5. Experimental Methods

Based on the typical chemical compositions of the raw materials, four ore blending schemes were designed for pellet preparation tests, with the detailed proportions presented in Table 4. To ensure the chemical stability of the finished pellets and their adaptability to blast furnace burden structures, the target SiO_2_ content of the finished pellets was fixed at 6.5% ± 0.15% across all schemes. Bentonite was employed as the binder, and its addition ratio was dynamically adjusted according to the balling performance of each blended mixture, so as to secure adequate green ball strength and thermal stability.

Pelletizing tests were conducted on a total weight basis of 5 kg per batch. The three iron ore concentrates were first mixed for 3 min, followed by the addition of a predetermined proportion of bentonite and further mixing for another 3 min. Distilled water was then added to adjust the moisture content of the mixture to 7.0 ± 0.5%, and the blending continued for an additional 5 min. The homogenized mixture was subsequently fed into a disc pelletizer with a diameter of 1 m, an inclination angle of 47°, and a rotation speed of 23 r/min for green ball preparation. The green balls were manually screened, and those with a size fraction of 10–16 mm were selected as qualified green balls. Ten randomly selected green balls were used for compressive strength, drop strength, and decrepitation temperature tests. An additional approximately 500 g of qualified green balls was dried in a constant-temperature oven at 105 °C ± 5 °C for 6 h to determine the moisture content of the dried balls. The remaining qualified green balls were dried under the same conditions and subsequently subjected to firing tests in a horizontal tube furnace to determine the optimal balling and firing regimes. The roasting temperatures in this study were determined according to the specific requirements of each experimental scheme, and were not fixed at a single value. The corresponding temperature regimes (including target temperature, heating rate, and holding time) for each test will be provided in the relevant discussions below. The cold compressive strength and metallurgical properties of the finished pellets were determined in accordance with GB/T 14201-2018 [13].

## 3. Results and Discussion

### 3.1. Particle Size Distribution of the Blends

According to the preset blending schemes such as Table 4, the iron ore concentrates were thoroughly mixed in designated proportions, and the particle size distributions of the resulting blends were characterized by laser diffraction analysis. The results are presented in Figure 3.

As illustrated in the figures, the particle size distribution of the blended mixture underwent a marked transformation as the PFC1 addition increased from 0% to 30%. Specifically, the D50 value exhibited a gradual increase, recorded as 23.5 μm, 25.2 μm, 26.8 μm, and 28.5 μm, respectively. This trend indicates an overall coarsening of the particle size. On the other hand, the width of the particle size distribution (PSD) also widened considerably. In the absence of PFC1 (0% addition), the mixture exhibited the highest peak intensity and the narrowest distribution range, reflecting superior particle size uniformity. In contrast, at a PFC1 addition of 30%, the peak intensity dropped to its minimum, accompanied by a pronounced right-side tailing in the 100–1000 μm range, suggesting a substantial increase in coarse particle content and a significant broadening of the PSD. These changes are primarily attributable to the inherently coarse nature of PFC1, whose introduction diluted the fine-sized fraction of the original mixture, thereby leading to overall particle coarsening. From the perspective of particle size gradation, an appropriate proportion of coarse particles can serve as nucleation cores for green pellets, which is beneficial for enhancing bulk density and bed permeability, thereby improving firing efficiency and pellet strength. However, excessive PFC1 addition results in an insufficiency of fine particles, which diminishes the capillary and bonding forces among particles, consequently reducing the ballability and green pellet strength. Moreover, an overabundance of coarse particles may induce internal stress concentration within the green pellets, exerting an adverse effect on their thermal stability.

Therefore, an optimal size grading should achieve a balance between the skeletal support provided by coarse particles and the binding and filling functions furnished by fine particles. When substituting a portion of fine concentrates with PFC1, the blending ratio should be controlled within an appropriate range in conjunction with the optimization of pelletizing process parameters. This ensures that the blend contains sufficient coarse particles to promote packing while maintaining adequate fines to guarantee balling performance, thereby realizing a rational particle size distribution [14] and optimized pelletizing behavior.

### 3.2. Green Ball Properties

Green ball properties are key indicators for evaluating pelletizing quality, directly reflecting the balling characteristics of the blended mixtures and the effectiveness of the binder. They mainly include moisture content, drop strength, compressive strength, decrepitation temperature, and dry ball strength. To systematically investigate the effect of PFC1 blending ratio on green ball quality, the pelletizing time was fixed at 10 min, and the green ball properties under different PFC1 ratios were tested. Among these indices, drop strength and compressive strength characterize the impact resistance and compression deformation resistance of green balls, respectively; decrepitation temperature reflects the thermal shock resistance during drying; and dry ball strength is directly related to the structural integrity of pellets during subsequent firing. All measurements were carried out in accordance with standard industrial methods, with each test repeated three times and the average values reported as the final results. The detailed data are presented in Table 5.

As shown in Table 5, the moisture content of green balls increased gradually from 8.4% to 9.3% with increasing PFC1 proportion from 0% to 30%. This increase is primarily attributed to the inherently coarse particle size and low specific surface area of PFC1, which require more water to lubricate particle surfaces and enhance capillary forces, thereby ensuring smooth balling process. The bentonite dosage exhibited a trend of initially increasing and then decreasing: it was 2.8% at both 10% and 20% PFC1 proportions, slightly lower than the 3.2% used in the baseline scheme, and further decreased to 2.4% at 30% proportion, indicating that an appropriate addition of PFC1 can reduce bentonite consumption and contribute to savings in binder costs.

In terms of green pellet strength, the drop strength and compressive strength across all blending ratios ranged from 4.0 to 5.9 times/0.5 m and 16.2 to 20.1 N, respectively, both meeting the fundamental requirements for industrial production [15] (see Table 6 for industrial pellet specifications). It should be noted, however, that industrial-scale pelletizing discs possess significant size advantages over laboratory equipment, resulting in higher rolling efficiency and consequently allowing for a reduction in bentonite dosage. In contrast, due to scale limitations of laboratory apparatus, a higher bentonite addition is typically required to achieve green pellet properties comparable to those of commercial pellets.

Experimental results indicate that the optimal comprehensive green pellet strength was achieved at a PFC1 ratio of 10%, with a drop strength of 5.6 times/0.5 m and a compressive strength of 20.1 N, representing improvements of 40% and 24%, respectively, over the baseline case. When the ratio increased to 20%, the compressive strength declined to 16.9 N, essentially matching the baseline level. Upon further increasing the ratio to 30%, although the compressive strength recovered to 19.3 N, the dried pellet strength continued to decrease to 42.4 N, marking a 26.3% reduction compared to the 10% ratio. This demonstrates that excessive PFC1 incorporation exerts an adverse effect on dried pellet strength.

The decrepitation temperatures of all schemes exceeded 430 °C and exhibited an increasing trend with rising PFC1 proportion, reaching 500 °C at 30% proportion. This improvement can be attributed to the relatively coarse particle size and high capillary water migration rate of PFC1 (13.33 mm/min, as shown in Table 3), which facilitate rapid moisture removal during the drying stage of green balls, thereby effectively reducing the risk of decrepitation and enhancing thermal stability. However, the dry ball strength decreased continuously with increasing PFC1 proportion, from 51.3 N in the baseline scheme to 42.4 N at 30% proportion, corresponding to a reduction of approximately 17.4%. This deterioration is primarily ascribed to the fact that PFC1 is a hematite concentrate, which lacks the consolidation–strengthening effect derived from the generation of active secondary Fe_2_O_3_ during magnetite oxidation. Moreover, the increased content of coarse particles results in fewer contact points and reduced number of bonding bridges among particles, leading to insufficient structural compactness of the dried balls and consequently weakening the dry ball strength.

### 3.3. Preheating and Firing Tests

To systematically investigate the effects of firing parameters on pellet quality, the green balls prepared in Section 2.2 were thoroughly dried and then subjected to preheating and firing tests in a horizontal tube furnace. The study focused on the effects of key thermal parameters, including preheating temperature, preheating time, sintering temperature, and holding time, on the metallurgical properties of preheated and finished pellets, such as compressive strength, reducibility, and reduction swelling index. The optimal firing regime for pellets with varying PFC1 blending ratios was determined to provide data support and process guidance for industrial trial production.

#### 3.3.1. Effect of Preheating Temperature

To investigate the effect of preheating temperature on the strength of preheated pellets, the preheating time was fixed at 8 min, and the green balls were preheated at different temperatures. The preheating stage followed a stepwise heating regime: the temperature was raised from room temperature successively to 200 °C with a holding time of 2 min, 400 °C for 6 min, and 700 °C for 7 min, and then heated at a rate of 10 °C/min to 850, 900, 950, and 1000 °C, respectively, with a holding time of 8 min at each target temperature. The entire preheating process was conducted under an air atmosphere with a flow rate of 2 L/min. After preheating, the samples were removed from the furnace tube, cooled in air to room temperature, and then subjected to compressive strength testing. The results are presented in Figure 4.

Figure 4 illustrates the variation of cold compressive strength (CCS) of preheated pellets with preheating temperature (850–1050 °C) at different hematite concentrate (PFC1) blending ratios (0%, 10%, 20%, and 30%). Overall, increasing the preheating temperature significantly enhanced the CCS across all experimental groups, which is primarily attributed to the oxidation of magnetite and the subsequent recrystallization and interconnection growth of Fe_2_O_3_ crystallites. However, the strengthening behavior varied considerably with the PFC1 blending ratio. In the low-temperature range (850–900 °C), the recrystallization kinetics of Fe_2_O_3_ were relatively sluggish, and the externally added hematite particles temporarily behaved as an inert phase that interfered with the initial consolidation among magnetite particles; consequently, the CCS values of the 10% and 30% PFC1 groups were slightly lower than those of the baseline group. As the temperature increased to the intermediate range (950–1000 °C), the intensified oxidation reaction and enhanced solid-state diffusion led to a rapid increase in the strength of all samples.

At the highest tested temperature of 1050 °C, the differences among the groups became most pronounced. Although the data for the baseline and 30% PFC1 groups were recorded only up to 1000 °C in the present experimental design, the 20% PFC1 group exhibited exceptional high-temperature strengthening potential, with its CCS surging to nearly 900 N·P^−1^—substantially exceeding the approximately 720 N·P^−1^ observed for the 10% PFC1 group at the same temperature. This can be ascribed to the fact that the 20% hematite addition provided optimal heterogeneous nucleation sites, promoting efficient synergistic interconnection growth between the externally added hematite crystallites and the in-situ generated active Fe_2_O_3_ crystallites derived from magnetite oxidation, thereby constructing a dense Fe_2_O_3_ crystal-bridge network with high bonding strength. In contrast, the 30% blending ratio may have altered the exothermic oxidation balance or the pore evolution pathway, thereby suppressing the formation of an ideal interconnection structure.

Furthermore, extending the preheating time provided more favorable kinetic conditions for solid-state diffusion and the development of sintering necks, leading to a continuous improvement in the densification and mechanical strength of the pellets. However, the attenuation of the strength increment within the same extended time interval indicated that the recrystallization process was progressively transitioning from a rapid growth stage to a slow and stable stage. Notably, in the systems with lower PFC1 blending ratios, the active Fe_2_O_3_ generated from magnetite oxidation dominated the consolidation process, rendering the strengthening effect of prolonged preheating more pronounced. Conversely, in the systems with higher PFC1 blending ratios (e.g., 30%), where hematite predominated and the driving force for consolidation was relatively weak, the improvement in strength achieved solely by extending the preheating time was considerably limited. In summary, within the parameter range investigated, blending 20% hematite concentrate (PFC1) combined with a preheating temperature of 1050 °C represents the optimal processing condition for maximizing the mechanical strength of magnetite–hematite composite pellets.

#### 3.3.2. Effect of Preheating Time

To investigate the effect of preheating time on the strength of preheated pellets, preheating tests were conducted in a horizontal tube furnace, and the results are presented in Figure 5. The preheating stage followed the same stepwise heating regime as described in Section 3.3.1. After reaching 1000 °C, the samples were held for 4 min, 6 min, 8 min, and 10 min, respectively. Upon completion of preheating, the samples were removed from the furnace tube, cooled in air to room temperature, and subsequently subjected to compressive strength testing.

As shown in Figure 5, the preheated pellet strength exhibited an overall increasing trend with increasing preheating time under different PFC1 blending ratios. Prolonging the preheating time provided more favorable kinetic conditions for solid-state diffusion among particles and the recrystallization interconnection of Fe_2_O_3_, allowing the contact points between adjacent particles to gradually develop into sintering necks and grow continuously. As a result, the internal porosity of the pellets decreased while the degree of densification increased, giving rise to a continuous improvement in macroscopic compressive strength. Furthermore, within the same extended time interval, the attenuation of the strength increment indicated that the recrystallization process transitioned progressively from a rapid growth stage to a slow and stable stage. The extent of strength enhancement with preheating time varied among different PFC1 blending ratios. In low-PFC1 schemes, active Fe_2_O_3_ generated from magnetite oxidation effectively promotes consolidation, rendering the positive effect of preheating time more pronounced. In contrast, the 30% PFC1 scheme exhibits an anomalous “early rise followed by stagnation” trend: although its initial strength is significantly higher than that of other schemes, it plateaus or even slightly declines after 8 min. This behavior implies that excessive PFC1 may induce internal structural defects or impede further consolidation due to the predominance of hematite and a weaker driving force for bonding. The underlying mechanism warrants further elucidation through thermogravimetric–differential thermal analysis (TG-DTA).

#### 3.3.3. Changes in Properties of Fired Pellets

Roasting temperature and holding time are key thermal parameters governing the degree of consolidation of pellets. To systematically investigate their effects on the properties of fired pellets, single-factor experimental schemes were designed. The sintering temperature was varied at 1160 °C, 1190 °C, 1220 °C, and 1250 °C with a fixed holding time of 8 min, while the holding time was varied at 9 min, 12 min, 15 min, and 18 min with a fixed sintering temperature of 1220 °C. The firing tests were conducted in a horizontal tube furnace. The green balls were first preheated according to the regime described in Section 3.3.2, and subsequently fired at the designated temperature for the prescribed holding time. The furnace atmosphere was maintained as air with a flow rate of 2 L/min throughout the firing process. For each scheme, 60 fired pellets were randomly selected and tested, and the average value was reported as the final strength. The results are presented in Figure 6a,b.

The experimental results indicated that all blending schemes with 10–30% PFC1 exhibited superior fired pellet properties compared to the baseline scheme without PFC1, suggesting that an appropriate introduction of hematite concentrate is beneficial for improving the pellet firing behavior. However, the schemes with 10–20% PFC1 showed noticeably better firing performance than that with 30% PFC1, indicating that there exists an optimal PFC1 blending range, beyond which the beneficial effect of magnetite oxidation on consolidation would be weakened by an excessively high hematite proportion. Based on the firing results of all blending schemes (0–30% PFC1), the optimal sintering temperature was approximately 1220 °C, and the appropriate holding time ranged from 9 to 15 min. Specifically, for the schemes containing 10–20% PFC1, the optimal holding time was 9–12 min, under which the fired pellet strength met the requirements for blast furnace burden. When the PFC1 proportion was increased to 30%, however, the holding time needed to be extended to 15 min to compensate for the inferior consolidation kinetics of the hematite-dominated system [16], thereby ensuring adequate compressive strength of the fired pellets.

Increasing the roasting temperature from 1160 °C to 1250 °C at a fixed holding time, or extending the holding time from 9 min to 18 min at a fixed temperature, both significantly improved the compressive strength of fired pellets for all blending schemes. This improvement is fundamentally attributed to the accelerated grain growth and recrystallization interconnection of hematite particles at higher temperatures and longer holding times, which promoted the full development of sintering necks and the densification of pellets. For the magnetite-containing baseline and low-PFC1 schemes, the magnetite concentrate first oxidized to Fe_2_O_3_, and the newly formed Fe_2_O_3_ possessed high surface activity and large diffusion coefficients, providing a stronger driving force for recrystallization. Moreover, the volume expansion associated with oxidation helped fill pores and enhance densification. In addition, the relatively broad particle size distribution allowed fine particles to fill the interstices among coarse particles, increasing contact areas and diffusion pathways; the higher bed porosity facilitated inward O_2_ transport to promote oxidation; and the larger specific surface area provided higher surface free energy, lowering the nucleation barrier for recrystallization. These three factors collectively promoted consolidation from both thermodynamic and kinetic perspectives [17]. The elevation of sintering temperature not only increased the equilibrium driving force for recrystallization but also enhanced diffusion coefficients and reaction rates, while the prolongation of holding time ensured sufficient completion of the consolidation reactions. The synergistic effect of these two thermal parameters ultimately led to a substantial improvement in the compressive strength of the fired pellets.

#### 3.3.4. Metallurgical Properties of Fired Pellets

The reducibility of pellets is a critical metallurgical property that determines the fuel rate of blast furnaces. And directly determines the utilization efficiency of reducing gas and the coke rate. Therefore, systematically elucidating the influence of PFC1 addition on the reducibility and reduction swelling index of pellets is a fundamental prerequisite for evaluating their applicability in blast furnace smelting.

Within the thermal balance system of blast furnace ironmaking, the indirect reduction of iron oxides (Equation (5)) exhibits a mildly exothermic characteristic, which contributes to reducing the external heat demand of the process. In contrast, the direct reduction reaction (Equation (6)) is a strongly endothermic process, accounting for a considerable proportion of the total heat expenditure in the blast furnace.

(5) $${\mathrm{Fe}}_{2}O_{3}+\;3\mathrm{CO}=2\mathrm{Fe}\;+\;3{\mathrm{CO}}_{2}\;\;\;\Delta H\;=-32.4\;\mathrm{kJ}/\mathrm{mol}$$

(6) FeO + C=Fe + CO    ΔH =+152.2 kJ/mol

Since iron in the pellets is predominantly present in the form of Fe_2_O_3_, a higher degree of reduction facilitates more complete indirect reduction. The reaction heat released can effectively compensate for thermal losses associated with carbonate decomposition, slag and iron melting, and furnace heat dissipation, thereby reducing the consumption of coke as a heat source. Consequently, enhancing the indirect reduction ratio is not only a critical approach to improving the thermal efficiency of the blast furnace but also an important thermodynamic foundation for achieving low-carbon smelting and lowering the fuel ratio.

The reducibility (RI) was determined in accordance with GB/T 24189-2024 [18], which evaluates the extent and rate of oxygen removal from iron ores under conditions simulating the reduction zone of a blast furnace. The tests were conducted isothermally at 900 °C using a reducing gas mixture of CO and N_2_ with a CO volume fraction of 30% for a duration of 180 min. The reducibility was expressed as the degree of oxygen loss from the samples. The reduction swelling index (RSI) was measured following GB/T 13240-2018 [19]. The pellets were reduced at 900 °C in a CO–N_2_ gas mixture, and the pellet volumes before and after reduction were determined separately. The RSI was calculated using the following equation: RSI = (V_1_ − V_0_)/V_0_ × 100%, where V_0_ and V_1_ are the pellet volumes before and after reduction, respectively. Both tests were performed in a reduction furnace with a gas flow rate of 15 L/min. Three parallel samples were tested for each scheme, and the average values were reported as the final results. The test results are presented in Figure 7a,b.

The RI of finished pellets increased continuously with increasing PFC1 proportion from 0% to 30%, reaching 66.4% at 30% PFC1, representing an improvement of approximately 27% over the baseline scheme (52%), indicating that the addition of PFC1 significantly enhanced the reducibility of pellets. Meanwhile, the RSI showed a slight increase, but remained below 15% for all schemes (maximum 12.44%), meeting the requirements for blast furnace operation.

The improvement in reducibility is mainly attributable to three factors. First, PFC1 is a hematite concentrate, which possesses a higher initial oxidation state and a shorter reduction path compared with magnetite, making it thermodynamically more reducible. Second, the coarser particle size of PFC1 increased the porosity of the fired pellets, providing more channels for reducing gas diffusion, enlarging the gas–solid reaction interface, and thus improving the kinetic conditions. Third, with increasing PFC1 proportion, the mineralogical composition of the pellets gradually transitioned from a magnetite-dominant to a hematite-dominant type, which is kinetically more favorable for reduction.

The slight increase in RSI is attributed to two factors: the decreased magnetite proportion weakened the pore-healing effect caused by its oxidation-induced volume expansion, and the increased porosity facilitated the release of lattice distortion stress during reduction [20]. Nevertheless, the RSI values of all schemes were within the controllable range (≤12.44%) [21], posing no significant risk to the permeability of the blast furnace burden column.

### 3.4. Pot-Grate Test

To validate the reliability and industrial applicability of the laboratory-scale findings, pot-grate tests were conducted in a commercial grate-kiln production line. Green balls were prepared under a fixed pelletizing time of 8 min for each blending scheme, and those with a size fraction of 10–16 mm were screened and collected in batches of 3 kg. The green balls were loaded into dedicated stainless steel baskets and fed into the grate-kiln system along with the normal production material flow. During the pot-grate tests, the temperature of the grate preheating zone was maintained at 1000 °C, while the rotary kiln firing zone was set at 1250 °C. After the pellets had passed through the firing and cooling stages in the rotary kiln, the baskets were retrieved from the annular cooler. The compressive strength of the finished pellets was determined in accordance with ISO 4700:2015, with 60 randomly selected pellets tested for each scheme and the average value reported as the final result. The results are presented in Figure 8.

The results showed that the compressive strength of finished pellets decreased gradually with increasing PFC1 proportion from 0% to 30%, but remained above 2700 N/pellet for all schemes, meeting the requirements for blast furnace burden. This indicates that within the tested blending range, the addition of PFC1 poses no quality risk to the pellet strength. The pot-grate tests further confirmed that under appropriate firing regimes (1220 °C, 9–15 min), all blending schemes with 0–30% PFC1 yielded qualified pellets satisfying the industrial requirements for blast furnace use.

## 4. Conclusions

(1) PFC1 is a typical hematite concentrate characterized by high iron grade and low harmful element content, making it an economically feasible alternative to conventional magnetite concentrates. However, its relatively coarse particle size and low specific surface area result in poor balling performance when used alone, necessitating blending with fine magnetite concentrates to achieve an appropriate particle size distribution.
(2) At PFC1 blending ratios of 10–20%, the green balls exhibited excellent drop strength (>4 times/0.5 m) and compressive strength (~18 N), along with a high decrepitation temperature (≥480 °C) and satisfactory dry ball strength, demonstrating the optimal overall balling performance. This range is therefore recommended as the preferred blending ratio for industrial production.
(3) With increasing PFC1 proportion, the compressive strengths of both preheated and finished pellets showed a decreasing trend. Nevertheless, under appropriate firing regimes (1220 °C, 9–15 min), the compressive strengths of finished pellets from all blending schemes exceeded 2700 N. The addition of PFC1 significantly improved the reducibility of pellets (RI increased from 52% to 66%), while the reduction swelling index remained within a controllable range (≤12.44%), indicating overall excellent metallurgical performance.
(4) It is recommended that the PFC1 blending ratio be controlled within 10–20% in industrial practice. The firing temperature may be appropriately reduced compared with that used for pure magnetite-based schemes. Furthermore, the incorporation of pretreatment processes such as high-pressure grinding rolls can further optimize the firing regime and reduce process energy consumption.

## Figures and Tables

**Figure 1:**
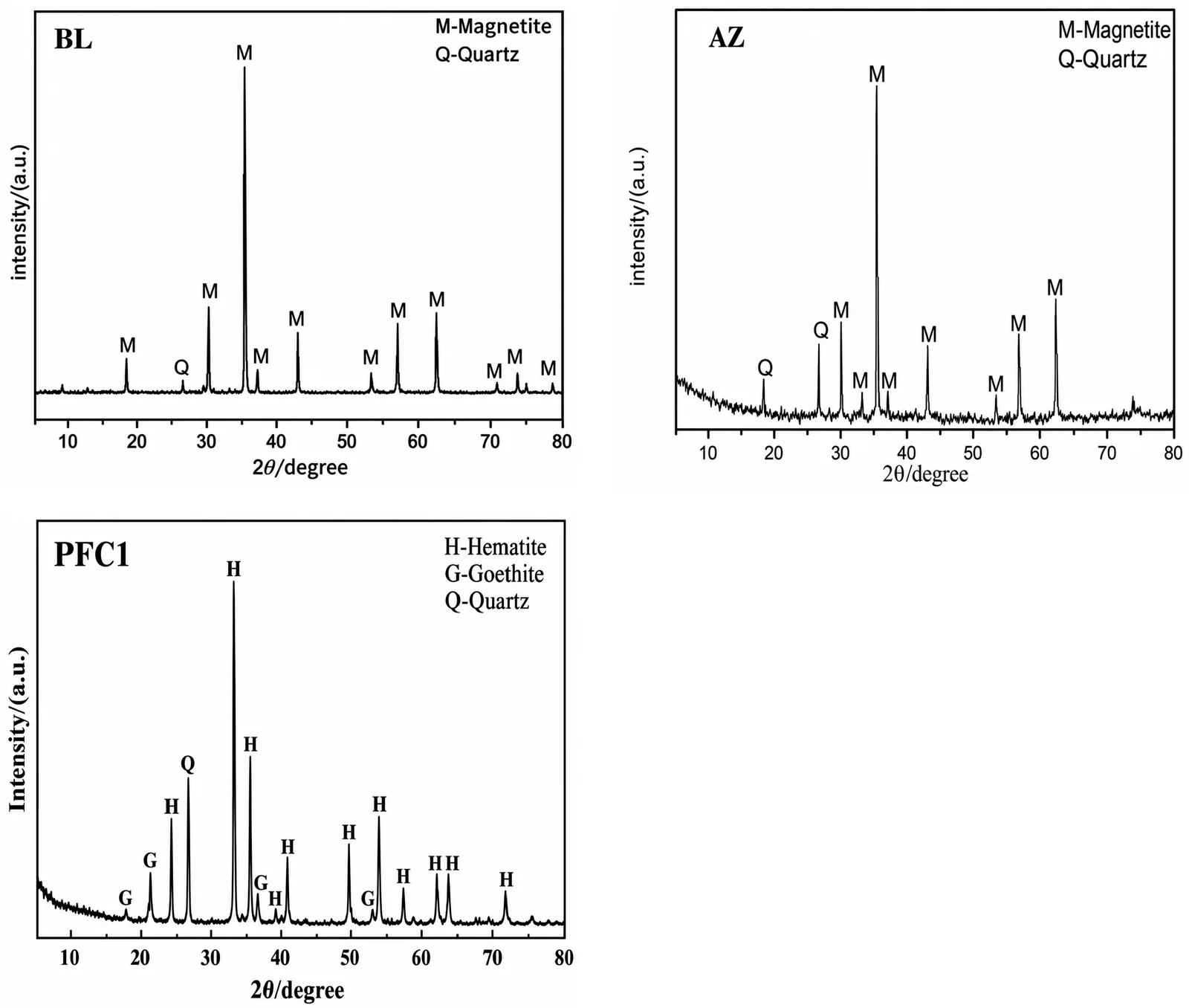
XRD analysis of three types of concentrates.

**Figure 2:**
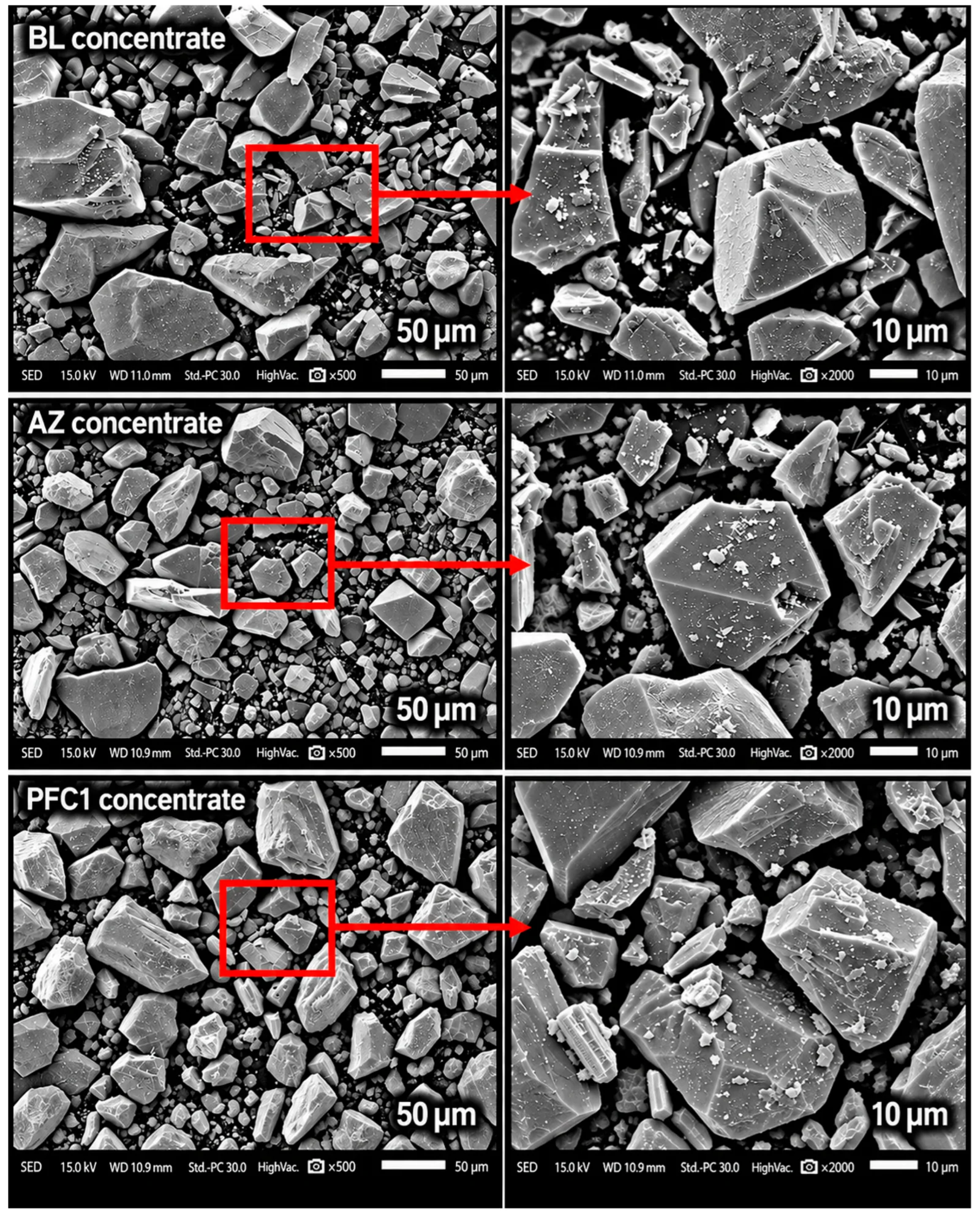
SEM microstructure analysis of three types of concentrates.

**Figure 3:**
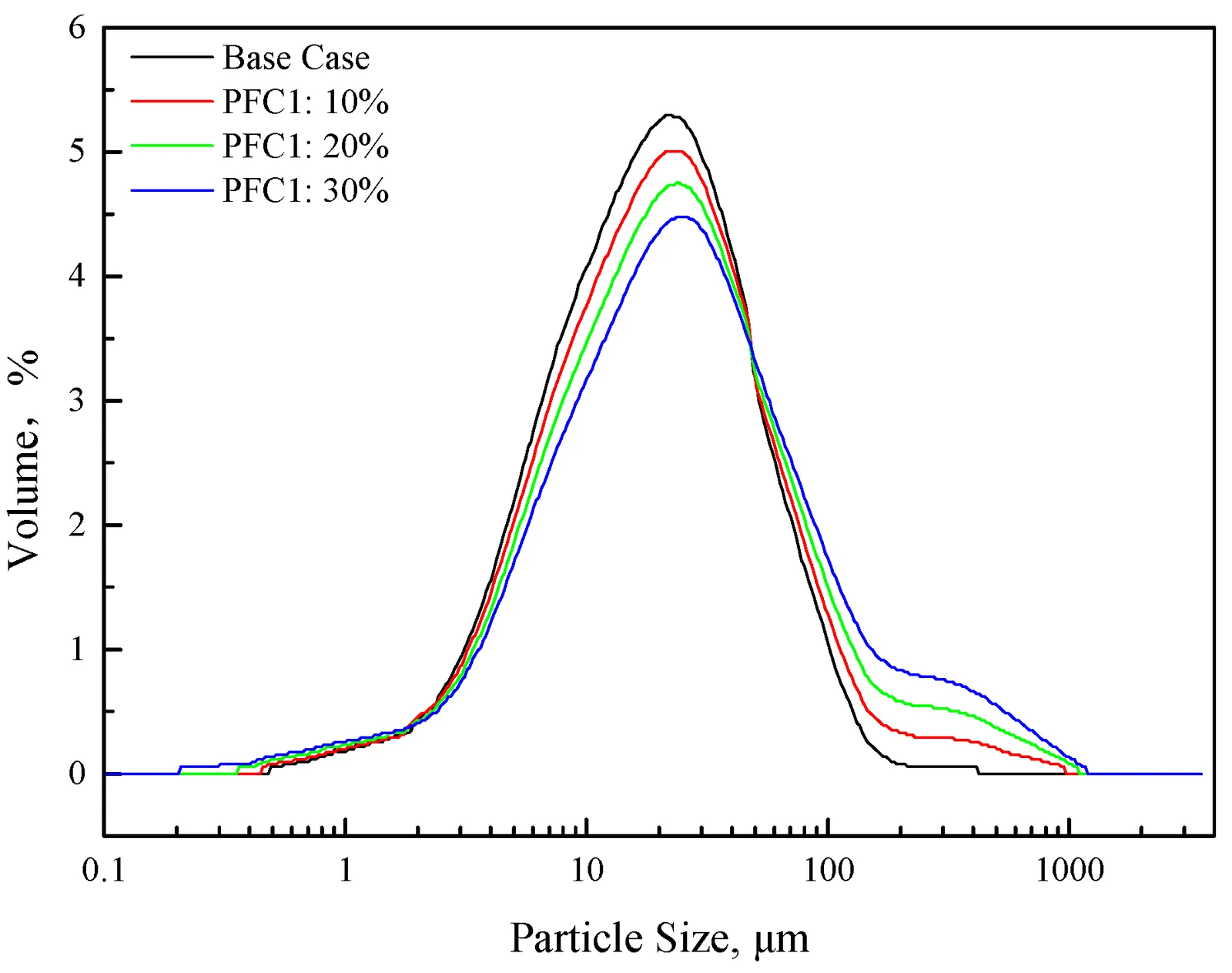
Particle size distribution of the mixed powder.

**Figure 4:**
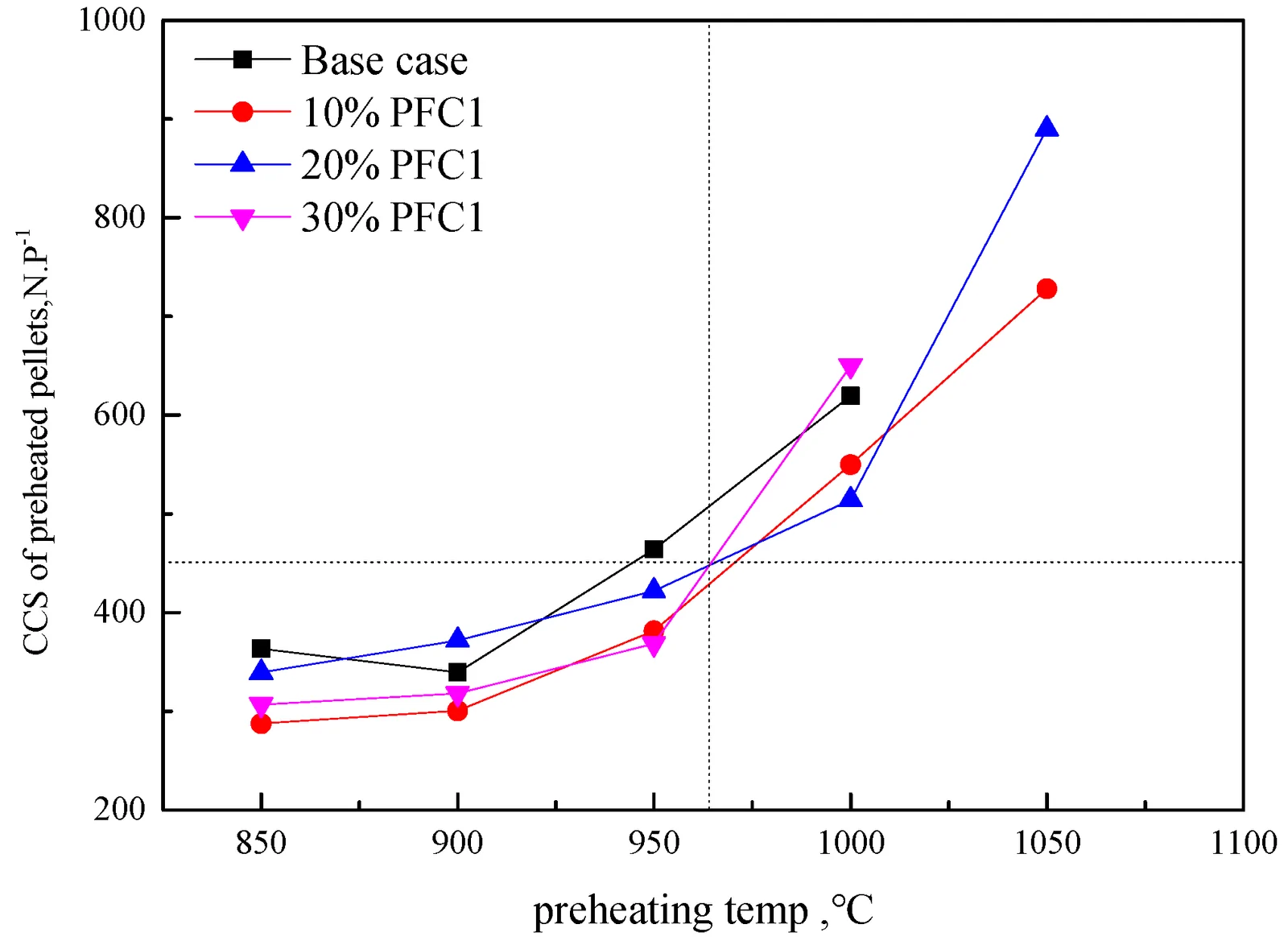
Variation of preheated pellet strength with temperature under different PFC1 ratios.

**Figure 5:**
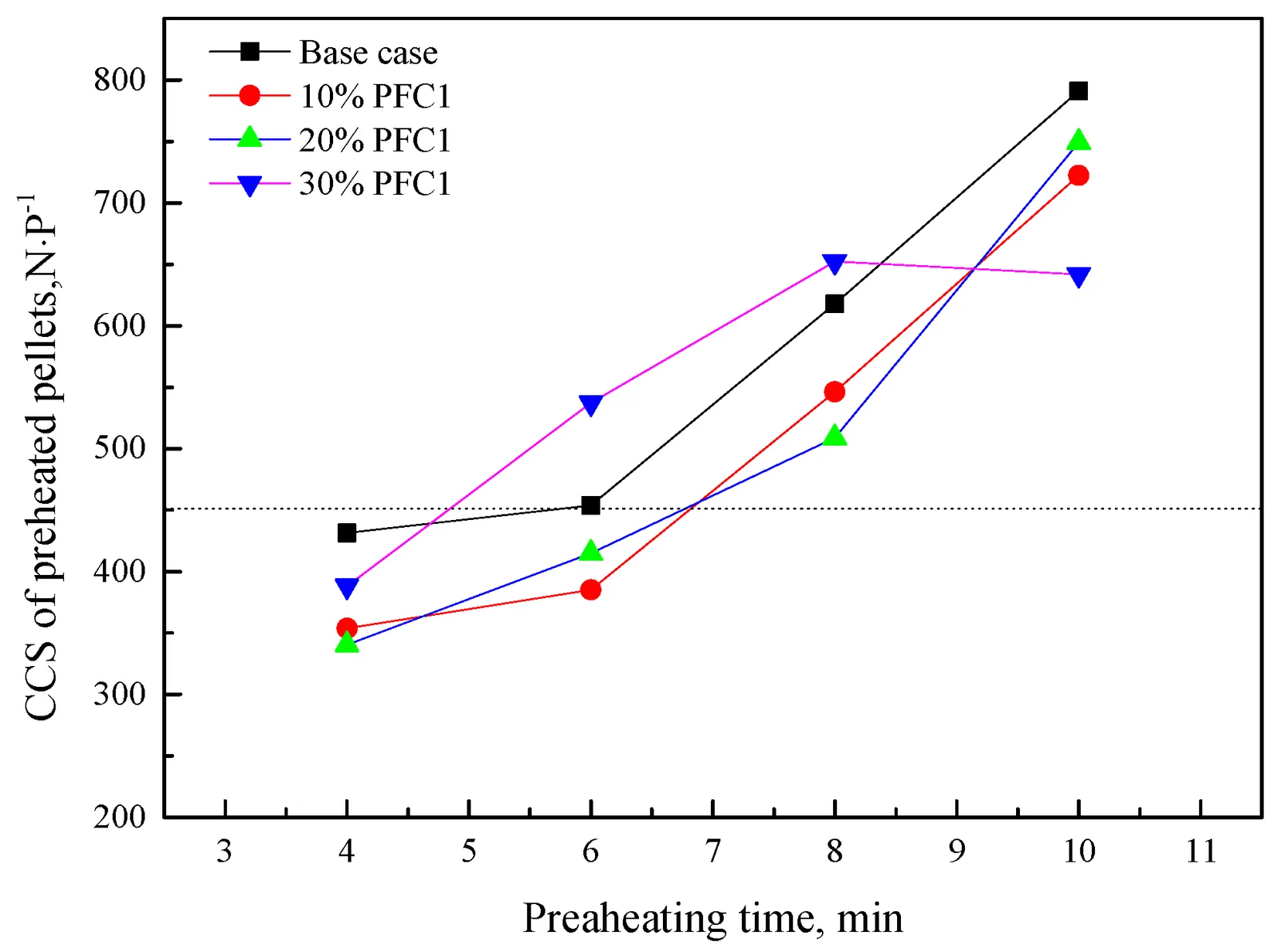
Preheated pellet strength vs. time for different PFC1 ratios.

**Figure 6:**
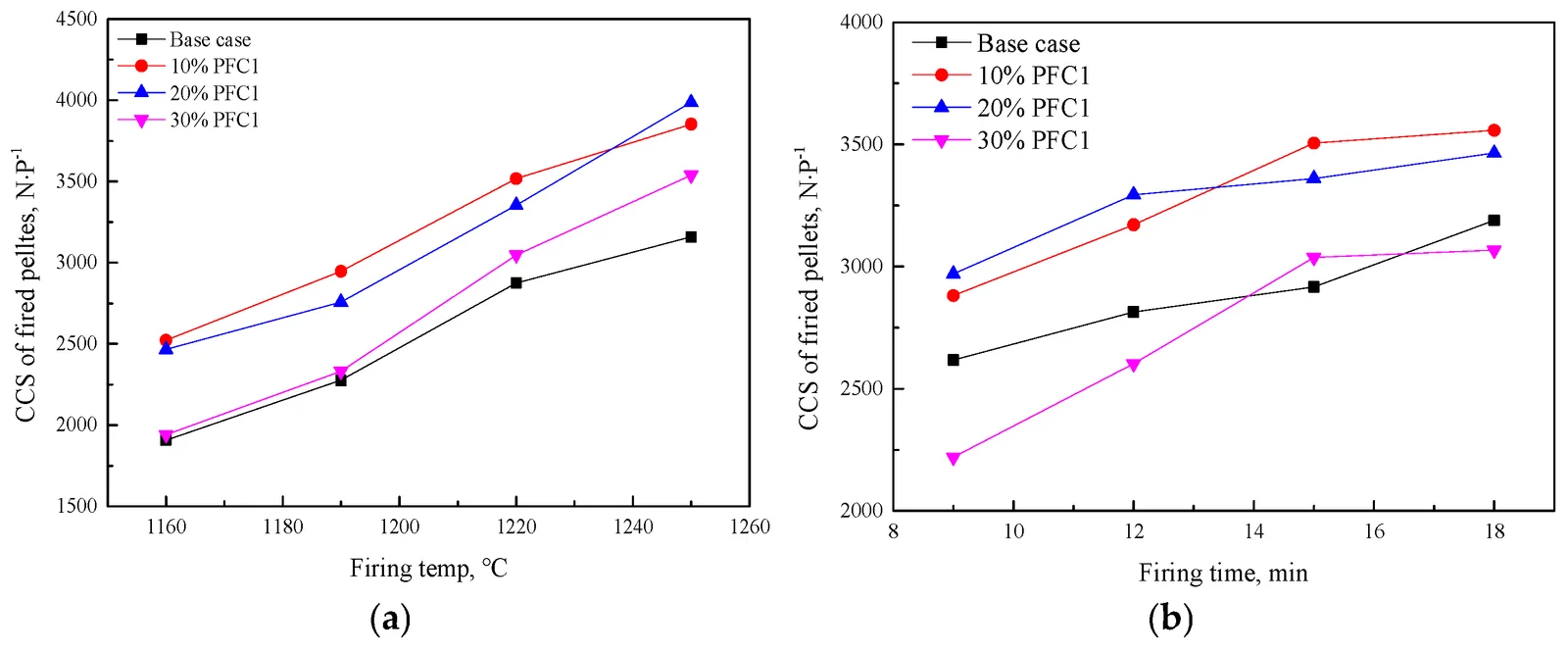
Effect of time and temperature on finished pellet strength with different PFC1 ratios. (**a**) Effect of Roasting Temperature on the Compressive Strength of Pellets; (**b**) Effect of Roasting Time on the Compressive Strength of Pellets.

**Figure 7:**
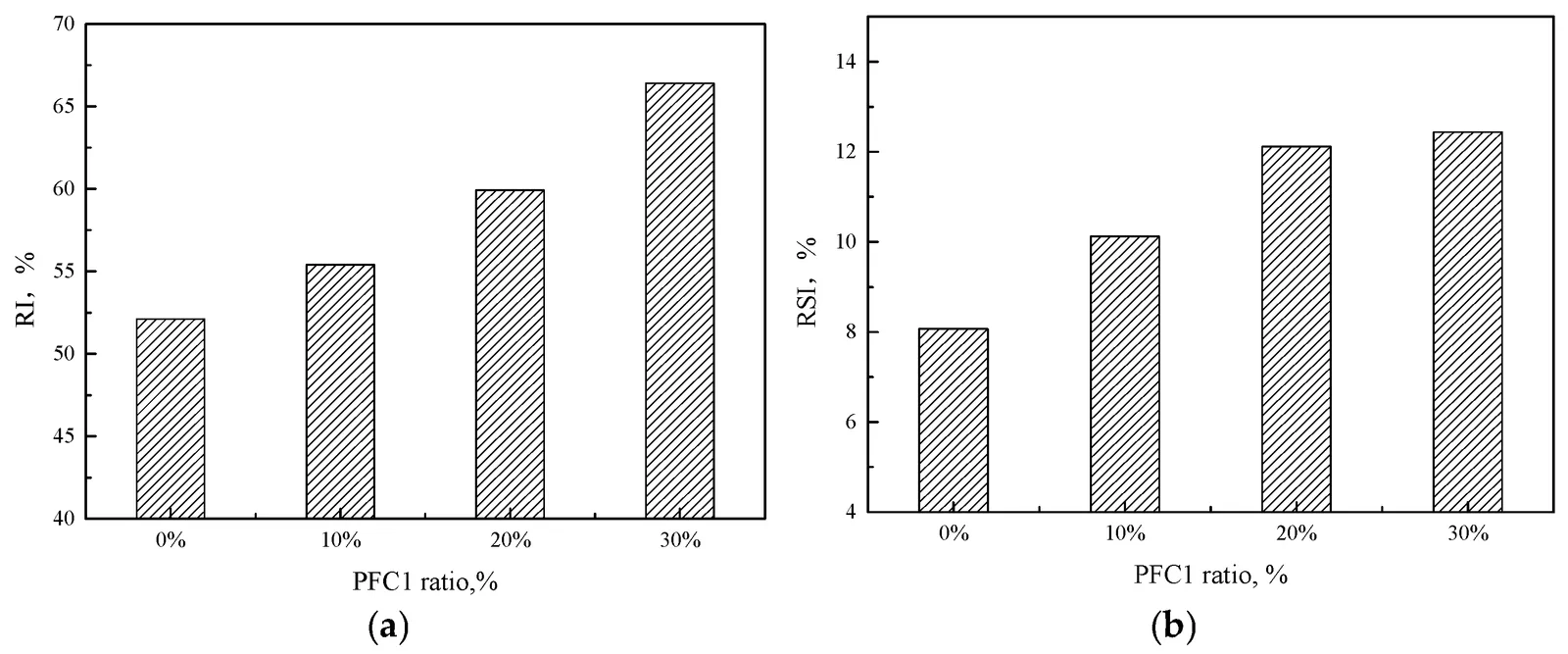
Metallurgical properties of finished pellets. (**a**) Effect of PFC1 Proportion on Pellet Reducibility; (**b**) Effect of PFC1 Proportion on the Reduction Swelling of Pellets.

**Figure 8:**
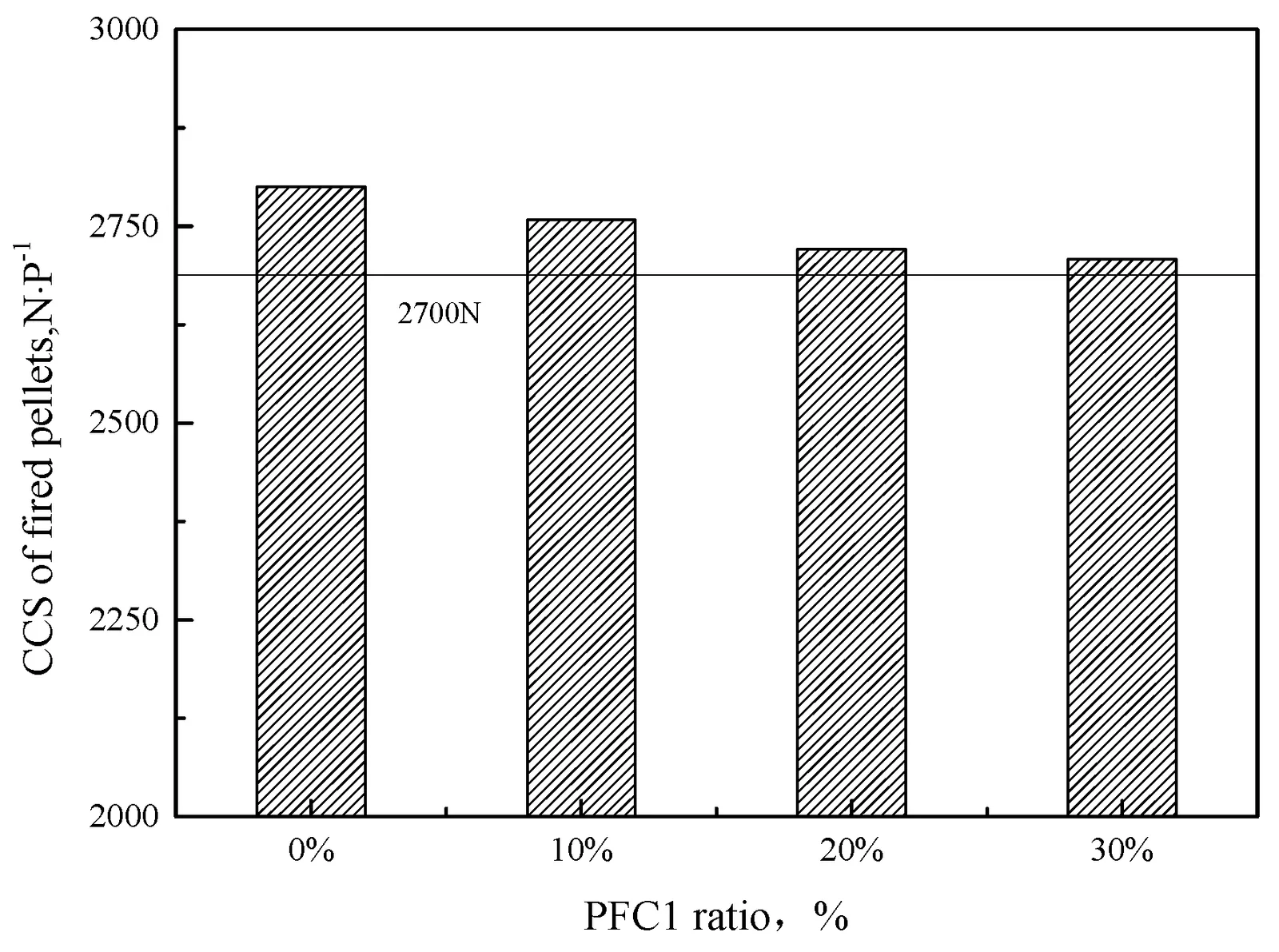
Variation in compressive strength of finished pellets after cage dropping.

**Table 1:** (**1**) Chemical composition of raw materials, %. (**2**) Performance of the bentonite.

(**1**)											
Raw Material	Fe	FeO	CaO	SiO_2_	Al_2_O_3_	MgO	P	S	Na_2_O	K_2_O	LOI
Bl Concentrate	69.19	30.25	0.27	2.14	0.32	0.50	0.025	0.260	0.091	0.056	−2.52
AZ Concentrate	65.30	27.3	0.16	8.17	0.25	0.42	0.045	0.050	0.150	0.170	−2.51
PFC1 Concentrate	62.45	1.07	0.05	5.52	1.06	0.09	0.048	0.036	0.012	0.006	2.92
Bentonite	1.86	1.23	2.10	67.13	13.40	1.58	0.084	0.042	2.360	2.230	5.37
(**2**)											
Type	Moisture %		CI %		SV mL·g^−1^		MAR % (2 h)	MBU g/100 g		Montmorillonite %	
Bentonite	9.40		44.5		4.5		242	23.5		53.20	

**Table 2:** Particle size distribution of three types of concentrates, wt%.

μm	+0.15 μm	0.074~0.15	0.045~0.074	0.038~0.045	−0.038	−0.074
BL Concentrate	0.46	6.24	19.58	13.20	60.52	93.3
AZ Concentrate	0.12	0.98	4.26	5.74	88.90	98.9
PFC1 Concentrate	18.66	22.24	16.84	8.20	34.06	59.1

**Table 3:** Physical properties of the three concentrates.

Type	BL Concentrate	AZ Concentrate	PFC1 Concentrate
SSA/cm^2^·g^−1^	1385	2567	847
δ-SSA	6.51	9.00	7.55
Bed porosity/%	64.13	65.71	54.43
δ-Bp	0.57	0.49	0.29
Capillary water migration rate/mm·min^−1^	8.93	6.75	13.33
δ-Cwm	0.13	0.08	0.11
Static ballability K_p_	0.11	0.16	0.26
δ-Sb	0.03	0.01	0.02

**Table 4:** Ore blending schemes for pellets, %.

Schemes	PFC1	BL	AZ	Bentonite	∑	P-T.Fe	P-SiO_2_
0	0	31.0	67.5	1.5	100	64.97	6.51
1	10	27.0	61.5	1.5	100	64.83	6.51
2	20	23.0	55.5	1.5	100	64.68	6.51
3	30	19.0	49.5	1.5	100	64.53	6.51

**Table 5:** Green pellet properties after pelletizing under four schemes.

PFC1 Concentrate	Bentonite, %	Green Pellet Moisture, %	Drop Number, Times/0.5 m	Green Pellet Compressive Strength, N	Cracking Temperature, °C	Dry Pellet Compressive Strength, N
Base-0%	3.2	8.4	4.0	16.2	430	51.3
10%	2.8	8.9	5.6	20.1	480	57.5
20%	2.8	9.3	5.9	16.9	480	49.5
30%	2.4	9.3	5.5	19.3	500	42.4

**Table 6:** Green pellet properties of industrial production.

PFC1 Concentrate	Bentonite, %	Green Pellet Moisture, %	Drop Number, Times/0.5 m	Green Pellet Compressive Strength, N	Cracking Temperature, °C	Dry Pellet Compressive Strength, N
0%	2.0	8.0	4.0	18.4	500	50.3

## Data Availability

The original contributions presented in this study are included in the article. Further inquiries can be directed to the corresponding author.
